# Molecular and cellular mechanisms of itch and pain in atopic dermatitis and implications for novel therapeutics

**DOI:** 10.1002/cti2.1390

**Published:** 2022-05-09

**Authors:** Shawn G Kwatra, Laurent Misery, Claire Clibborn, Martin Steinhoff

**Affiliations:** ^1^ 1500 Department of Dermatology Johns Hopkins University School of Medicine Baltimore MD USA; ^2^ Department of Dermatology University Hospital of Brest Brest France; ^3^ 97470 Pfizer Ltd Surrey UK; ^4^ 12295 Department of Dermatology and Venereology Hamad Medical Corporation Doha Qatar; ^5^ Translational Research Institute Academic Health System Hamad Medical Corporation Doha Qatar; ^6^ Dermatology Institute Academic Health System Hamad Medical Corporation Doha Qatar; ^7^ Department of Dermatology Weill Cornell Medicine‐Qatar Doha Qatar; ^8^ Qatar University, College of Medicine Doha Qatar; ^9^ 12295 Department of Dermatology Weill Cornell Medicine New York NY USA

**Keywords:** atopic dermatitis, cytokines, interleukins, neuroimmunology

## Abstract

Atopic dermatitis is a chronic inflammatory skin disease. Patients with atopic dermatitis experience inflammatory lesions associated with intense itch and pain, which lead to sleep disturbance and poor mental health and quality of life. We review the molecular mechanisms underlying itch and pain symptoms in atopic dermatitis and discuss the current clinical development of treatments for moderate‐to‐severe atopic dermatitis. The molecular pathology of atopic dermatitis includes aberrant immune activation involving significant cross‐talk among the skin and immune and neuronal cells. Exogenous and endogenous triggers modulate stimulation of mediators including cytokine/chemokine expression/release by the skin and immune cells, which causes inflammation, skin barrier disruption, activation and growth of sensory neurons, itch and pain. These complex interactions among cell types are mediated primarily by cytokines, but also involve chemokines, neurotransmitters, lipids, proteases, antimicrobial peptides, agonists of ion channels or various G protein–coupled receptors. Patients with atopic dermatitis have a cytokine profile characterised by abnormal levels of interleukins 4, 12, 13, 18, 22, 31 and 33; thymic stromal lymphopoietin; and interferon gamma. Cytokine receptors mainly signal through the Janus kinase/signal transducer and activator of transcription pathway. Among emerging novel therapeutics, several Janus kinase inhibitors are being developed for topical or systemic treatment of moderate‐to‐severe atopic dermatitis because of their potential to modulate cytokine expression and release. Janus kinase inhibitors lead to changes in gene expression that have favourable effects on local and systemic cytokine release, and probably other mediators, thus successfully modulating molecular mechanisms responsible for itch and pain in atopic dermatitis.

## Graphical abstract

In this article, we review molecules that mediate the symptoms of atopic dermatitis (AD), including cytokines that signal through the Janus kinase (JAK)/Signal transducer and activator of transcription (STAT) pathway and interact with the immune and nervous systems. We describe JAK inhibitors that are in development or approved to treat AD.

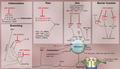

## Introduction

Atopic dermatitis (AD) is a chronic, relapsing, inflammatory skin disease that typically begins during childhood^1^; however, late‐onset or adult AD can also occur. AD prevalence is dependent on age and ranges from 2% to 15%, with about 20% of cases being moderate to severe.^2^

Pruritus and pain are hallmark symptoms of AD and have an adverse impact on quality of life.^3^ Patient‐reported data have established that itch and pain are the most burdensome and impactful symptoms of AD.^3^ Itch and pain contribute to impairment in work productivity and social dysfunction.^4^ Severe AD is associated with an increased risk of death, in particular from infection, possibly because of cutaneous or extracutaneous infection or immunosuppressive treatments.^5^

The pathophysiology of AD involves aberrant interactions among various cell types in the skin, and immune and nervous systems that are mediated by a host of immune‐related molecules including toll‐like receptors (TLRs) and cytokines, neurotransmitters and their cognate receptors and various other molecules involved in signal transduction that lead to itch and pain.^6^, ^7^ Understanding these pathways provides insight into rational therapeutic approaches to achieve disease and symptom control and potentially offers insight into preventive options when used as maintenance therapy. Cytokines are of particular interest because they are involved in cross‐talk between the immune and sensory systems. Several key cytokines signal through a family of Janus kinase/signal transducer and activator of transcription (JAK/STAT) molecules, making these an important class of drug targets.^8^

## Dysregulation of adaptive/innate responses in AD

Although the development of AD is not well understood, it likely involves initial interactions between the skin microbiome and the host innate immune response that further develops with the involvement of adaptive immunity. Patients with AD have abnormal innate and adaptive immune responses.^9^

Signalling through TLRs expressed on cells in the skin plays a key role in host innate immune response to pathogens. Thirteen TLRs have been identified, each reactive to components of specific types of pathogens. For example, TLR2 detects various components of gram‐positive bacteria.^6^ Keratinocytes express several TLRs, including TLR2, TLR3 and TLR5, which, upon activation by specific pathogens, leads to an innate proinflammatory response including production of the cytokines, tumor necrosis factor α (TNF‐α) and IL‐6.^6^ Dendritic cells (DCs) in the skin express most TLRs, and plasmacytoid DCs, which infiltrate the skin on injury, express TLR7 and TLR9 that recognise nucleic acids derived from bacteria, viruses and damaged cells.^6^ TLRs on DCs signal through myeloid differentiation primary response 88 (MyD88), activating protein 1 (AP‐1) and NFκB, resulting in increased expression of genes encoding proinflammatory cytokines and subsequent AD.^6^ TLR‐mediated innate immune activation in DCs also results in recruitment of macrophages and T cells, and AD subsequently occurs when excess inflammation is unresolved.^6^ Thus, TLRs are at the border of innate and adaptive immunity.^6^

The pathophysiology of AD is characterised by a prominent, abnormal Th2 immune response. Langerhans cells in the epidermis recognise foreign antigens and prime T cells as part of the host defence.^6^ Activation of TLR2 and production of IL‐4 induce keratinocytes to produce thymic stromal lymphopoietin (TSLP), which is a key regulator of the Th2 response. TLR2 and TSLP link the innate and Th2‐skewed adaptive immune responses in patients with AD, leading to worsening and persistence of the disease.^6^ TSLP also activates T cells, DCs and mast cells.^10^ TSLP promotes itch via the TSLP receptor by activating cutaneous sensory neurons that express transient receptor potential ankyrin 1 (TRPA1) ion channels.^11^ The long‐term and short‐term effects of TSLP on itch may be distinct, with acute effects involving activation of sensory neurons and chronic effects involving recruitment of various immune cells including macrophages and T cells.^11^

Deviant Th1, Th17 and Th22 responses are also involved to varying degrees in certain AD endotypes (e.g. paediatric and Asian subtypes).^12^ Th1‐mediated dysregulation has been implicated in the chronic form of AD.^2^ Abnormal TLR2 activation may also induce Th1 responses and TSLP production by keratinocytes, driving AD pathology by linking the innate immune response to pathogens at the skin barrier with abnormal Th2 adaptive immune responses.^6^ Th2/Th17 expression is especially prominent in African American patients with AD (Th1, Th17 and Th22).^13^

Some cytokines and receptors involved in the innate and adaptive immune response modulate the sensation of itch and pain in AD via TRP ion channels.^14^ In mast cell–deficient mice with transgenic expression of IL‐13 in skin, IL‐13 increases expression of TRPA1 ion channels in dorsal root ganglia (DRGs) and mast cells and induces itch.^15^ Recruitment of T cells to AD lesions, production of IL‐31 and subsequent activation of sensory neurons may drive and exacerbate itch.^16^ Sensory neurons that express transient receptor potential vanilloid (TRPV) 1‐4^17^, ^18^ and IL‐31 receptor also express TRPV1 and TRPA1 ion channels, thus linking T cell–mediated and sensory nerve–mediated itch.^19^ Neurons activated by cytokines, in turn, release neurotransmitters and neuropeptides that affect innate and adaptive immune cells.^20^ Thus, cytokines released from skin and immune cells have a direct impact on neurons via TRP ion channels, the activation of which produces itch.

## Cytokines in AD: role in itch, pain and skin barrier dysfunction

### Early events in AD

Key cytokines implicated and upregulated in AD include IL‐4, IL‐13, IL‐31 and others.^2^ Although AD is primarily a disease of Th2 dysregulation, with IL‐4, IL‐13, IL‐31 and TSLP being key players, cytokines such as interferon (IFN)‐γ (Th1), IL‐17, IL‐22 (Th17) and IL‐33 are also involved.^21^ Key cytokines that play a role in the early pathophysiology of itch, pain and disruption of the skin barrier in patients with AD are described below and summarised in Table 1.

**Table 1:** Key cytokines involved in atopic dermatitis

Cytokine	Cellular origin/stimulus	Cytokine receptor	Cell type(s) expressing the receptor	Overall function/AD MOA, itch, pain	References
IL‐4	ILC2, CD4^+^ T cells, eosinophils, basophils	IL‐4Rα, γC	Sensory neurons	Development of Th2 cells; ↓EDC molecules; ↑itch, IL‐31, MHC class II; activate sensory neurons	22, 24, 40, 41, 114, 141
IL‐13	Mast cells, basophils, eosinophils, ILC2, CD4^+^ T cells	IL‐4Rα, IL‐13rα1	Sensory neurons	Induce Th2 response; ↑IL‐31, TRPA1, branching, itch; ↓filaggrin; activate sensory neurons	2, 24, 40
IL‐22	Mast cells	IL‐10 family		↓Filaggrin, claudin‐1; ↑GRP, TSLP, IL‐33, itch	29, 30
IL‐31	T cells, DCs, Th2 cells, skin cells, eosinophils, basophils, mast cells	IL‐31R, OSMR	DRGs, sensory nerves, leukocytes, keratinocytes	↑NKB, itch, BNP, branching; ↓filaggrin; stimulate sensory neurons via TRPA1	19, 32, 35, 40, 80
TSLP	Keratinocytes/in response to proteases and bacteria	TSLPR, IL‐7Rα	Sensory nerves, ILC2	↑IL‐31, IL‐33, itch; activate sensory neurons via TRPA1, T cells, DCs, mast cells; stimulate ILC2; link innate and adaptive immune responses	2, 10, 11, 39, 50, 142
IL‐33	Keratinocytes, macrophages, DCs	IL‐33R, ST2	Astrocytes, mast cells, ILC2	↑IL‐4, IL‐13, IL‐33, TNF‐α, CXCL8, PGD2, itch	44, 49, 50
IL‐17	CD4^+^ T cells	IL‐17RA	Skin cells, ILC2	↑GM‐CSF	48, 50, 54
IL‐25	Keratinocytes	IL‐17RB	ILC2	↑IL‐13	49, 50
IL‐18	Keratinocytes, macrophages, DCs	IL‐18R	Th1 cells	↑IFN‐γ, ↑IL‐4, chemokines, inflammation, skin barrier dysfunction, skin pain	52
GM‐CSF	Keratinocytes, T cells, B cells, NK cells, monocytes/macrophages, fibroblasts, mast cells	GM‐CSFRα	Keratinocytes	Induces Th2 response	54

↓, decreased; ↑, increased; AD, atopic dermatitis; BNP, brain natriuretic peptide; DC, dendritic cell; DRG, dorsal root ganglion; EDC, epidermal differentiation complex; GM‐CSF, granulocyte‐macrophage colony‐stimulating factor; GM‐CSFR, granulocyte‐macrophage colony‐stimulating factor receptor; GRP, gastrin‐releasing peptide; IL, interleukin; ILC2, type 2 innate lymphoid cells; MOA, mechanism of action; NKB, neurokinin B; OSMR, oncostatin M receptor; PGD2, prostaglandin D2; ST2, stromal cell line; TNF, tumor necrosis factor; TRPA, transient receptor potential ankyrin; TSLP, thymic stromal lymphopoietin; TSLPR, thymic stromal lymphopoietin receptor.

### IL‐4

IL‐4 is produced by mast cells, type 2 innate lymphoid cells (ILC2), CD4^+^ T cells, eosinophils and basophils and facilitates Th2 cell development.^22^ In AD lesions, IL‐4 downregulates multiple genes involved in innate defence, including genes in the epidermal differentiation complex critical for epidermal barrier function.^21^ IL‐4 activates/sensitises IL‐4Ra on sensory nerve endings and induces JAK‐STAT signalling, thereby contributing to neuroinflammation and itch.^23^, ^24^

### IL‐13

IL‐13 is produced by basophils, mast cells and eosinophils.^25^ IL‐13 along with IL‐4 are major players in induction of the Th2 response and blocking expression of the barrier protein filaggrin, leading to skin barrier dysfunction.^2^ Sensory neurons are activated by IL‐4 and IL‐13, which sensitise small‐diameter sensory neurons to other inducers of itch, including histamine, chloroquine, TSLP, protease‐activated receptor 2 (PAR‐2), leukotrienes and IL‐31.^24^, ^26^, ^27^ IL‐13Ra1 is primarily responsible for the IL‐13–mediated role in itch, whereas IL‐13Ra2 is probably involved in neuroinflammation; its role in itch or pain is not yet clear.^28^

### IL‐22

IL‐22, which is increased in patients with AD, is produced by mast cells^29^ and downregulates expression of filaggrin and the tight junction protein claudin‐1.^30^ High levels of IL‐22 are also produced by circulating CD4^+^ and CD8^+^ T cells in patients with prurigo nodularis, which often presents as a manifestation of AD in African Americans.^31^ IL‐22 also upregulates expression of the pruritogenic peptide gastrin‐releasing peptide (GRP) in sensory neurons and expression of TSLP and IL‐33 in epithelial cells and increases itch and AD‐like disease in mice.^30^ Whether IL‐22–induced keratinocyte activation leads to the release of itch mediators is not yet clear.

### IL‐31 and oncostatin M

T cell–derived pruritogenic IL‐31 is upregulated in AD, and DRGs express high levels of the IL‐31 receptor, which signals through extracellular signal‐regulated kinase to induce itch.^16^, ^19^, ^32^ Other cell types that express IL‐31 include keratinocytes, eosinophils, basophils, mast cells, DCs and monocytes.^16^, ^33^ IL‐31 induces the expression of neurokinin B, which is required for IL‐31–mediated itch in DRG neurons.^34^ IL‐31 downregulates filaggrin expression.^35^ Consistent with a role for IL‐31 in itch, a phase 2 trial of nemolizumab, a humanised antibody targeting IL‐31 receptor A, demonstrated significant itch reduction in patients with moderate‐to‐severe AD.^36^ In addition, promising results have been seen with nemolizumab in prurigo nodularis.^37^

Oncostatin M (OSM), which is produced by dermal T cells and monocytes, is a highly upregulated cytokine in AD and is associated with chronic itch. OSM is a non‐canonical inducer of itch. Rather than activating sensory neurons, OSM sensitises neurons by increasing neural excitability and responses to pruritogens. The OSM receptor (OSMR; shared with the IL‐31 receptor) is expressed by natriuretic polypeptide B–positive itch‐selective neurons. OSMR knockout in DRG neurons decreases OSM‐mediated itch in mice, and pharmacologic inhibition of OSMR decreases itching in experimental inflammatory dermatitis in mice.^38^

### Thymic stromal lymphopoietin

In response to proteases and bacteria on the skin surface, keratinocytes produce cytokines including TSLP,^2^, ^39^ which promotes itch by directly activating sensory neurons.^40^ TSLP stimulates ILC2, which bridges the innate and adaptive immune response in AD and secretes type 2 cytokines including IL‐4, IL‐5 and IL‐13.^41^ Protease PAR‐2 activation stimulates release of TSLP from keratinocytes, which activates TSLP receptor (TSLPR) on sensory nerve endings, thereby inducing pruritus.^11^ Although clinical trials of antibodies against TSLP/TSLPR have been successful in treating asthma,^42^ but not yet in AD,^43^ the beneficial role of TSLP/TSLPR blockage in treating itch and AD cannot be fully excluded.

### IL‐33

IL‐33 is produced by keratinocytes, endothelial cells and immune cells; is upregulated in AD; and is an early ‘danger alarmin’ that activates the innate immune system.^44^ IL‐33 enhances expression levels of IL‐4 and IL‐13, which in turn stimulate synthesis of the itch‐producing IL‐31.^2^, ^44^ In addition, IL‐33 stimulates basophils to activate ILC2 via IL‐4, and both cytokines can also induce itch. Conversely, itch mediators such as IL‐31, substance P and brain natriuretic peptide (BNP) can also stimulate release of cytokines and chemokines that are involved in inflammation, barrier dysfunction, itch and pain.^16^, ^40^, ^45^, ^46^, ^47^

### IL‐17

IL‐17 is produced by CD4^+^ T cells, regulates the expression of chemokines by keratinocytes, exacerbates AD and is found to be significantly increased in the skin of African Americans with lesional AD.^13^, ^48^ Whether IL‐17 cytokine family members are involved in pain or itch in patients with AD is not clear yet.

### IL‐25

Stimulated keratinocytes secrete IL‐25, which is upregulated in AD lesions.^49^, ^50^ IL‐25 and IL‐33 induce secretion of type 2 cytokines (IL‐5, IL‐9 and IL‐13) from ILC2s, further perpetuating the pathophysiology of AD, including skin barrier dysfunction.^50^ Because IL‐25 and IL‐33 are involved in eosinophil recruitment, both cytokines may be involved in eosinophil‐associated itch in patients with AD.^51^

### IL‐18

IL‐18 is expressed by keratinocytes, is increased in patients with AD^52^ and contributes to inflammation and skin barrier dysfunction. IL‐18 increases IFN‐γ production and subsequent chemokine secretion by keratinocytes.^53^ Whether IL‐18 is associated with TSLP‐mediated itch from keratinocytes or pain is not known.

### Granulocyte‐macrophage colony‐stimulating factor

**Figure 1:**
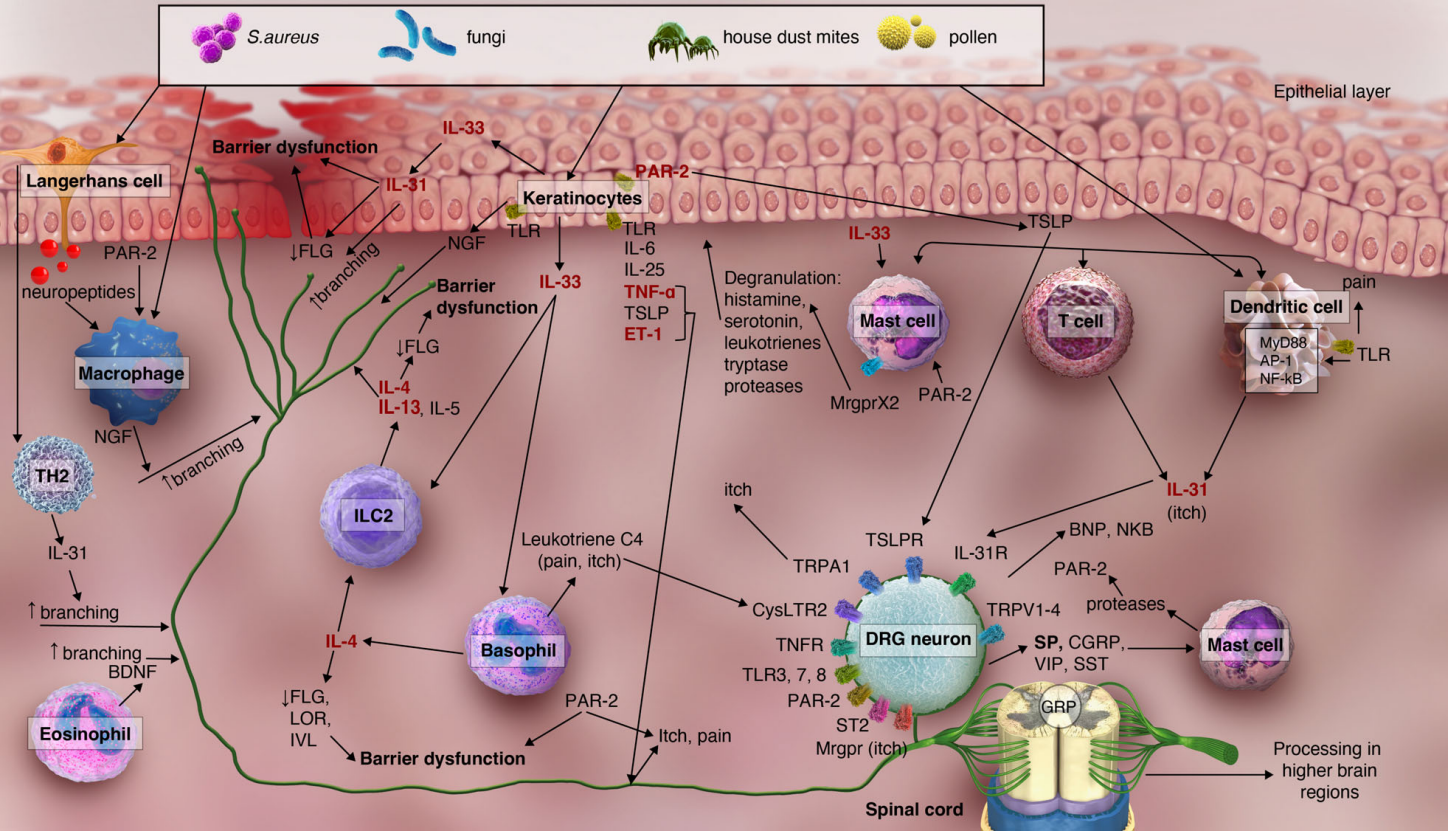
The cytokine network in atopic dermatitis with effects by and on various cell types, with a focus on neuroinflammation, itch and pain. Cytokines in red/bold text are upregulated in atopic dermatitis (AP‐1, activating protein 1; BDNF, brain‐derived neurotrophic factor; BNP, brain natriuretic peptide; CGRP, calcitonin gene‐related peptide; CysLTR2, leukotriene receptor; DRG, dorsal root ganglion; ET‐1, endothelin 1; FLG, filaggrin; GRP, gastrin‐releasing peptide; IL, interleukin; IL‐31R, IL‐31 receptor; ILC2, innate lymphoid cell 2; IVL, involucrin; LOR, loricrin; Mrgpr, Mas‐related G protein–coupled receptor; MrgprX2, Mas‐related G protein–coupled receptor X2; NGF, nerve growth factor; NKB, neurokinin B; PAR‐2, proteinase‐activated receptor 2; *S. aureus*, *Staphylococcus aureus*; SP, substance P; SST, somatostatin; ST2, IL‐33 receptor; TLR, toll‐like receptor; TNF, tumor necrosis factor; TNFR, TNF receptor; TRPA1, transient receptor potential ankyrin 1; TRPV4, transient receptor potential vanilloid 4; TSLP, thymic stromal lymphopoietin; TSLPR, thymic stromal lymphopoietin receptor; VIP, vasoactive intestinal peptide).

Granulocyte‐macrophage colony‐stimulating factor (GM‐CSF) is produced by keratinocytes in patients with AD at higher levels than keratinocytes in healthy controls.^54^ GM‐CSF plays a role in inducing the Th2 immune response in patients with AD, and its production may be mediated by the proinflammatory PAR‐2 and induced by IL‐17.^54^ Thus, in the early stages of AD pathology, multiple cytokines produced by various immune and skin cells not only mediate the inflammatory symptoms of AD but also drive itch, pain and skin barrier dysfunction (Figure 1). Thus, current literature indicates that GM‐CSF is primarily indirectly involved in itch or pain in AD.

## Cellular mediators of itch and pain in AD

Different cell types mediate longer‐term events in patients with AD and perpetuate itch and pain. Specialised cells such as Schwann cells in the peripheral nervous system and astrocytes or oligodendrocytes in the central nervous system (CNS) communicate with immune cells to mediate chronic itch and pain.^55^, ^56^ IL‐33 binds to its receptor expressed on astrocytes and induces production of proinflammatory cytokines via JAK2/STAT3 signalling, thus exacerbating itch.^57^ Intrathecal administration of the astrocyte inhibitor, l‐α‐aminoadipate, attenuates chronic itch.^58^

Glial cells also play a role in neuropathic pain. In the peripheral nervous system, satellite glial cells surround DRG cell bodies, are activated after nerve injury and play a role in the initiation and maintenance of neuropathic pain.^59^ Afferent pain signals lead to secretion of adenosine triphosphate (ATP), which induces satellite glia to produce matrix metalloproteinase 9 (MMP‐9), nerve growth factor (NGF) and ATP, which in turn activate DRG neurons and result in peripheral sensitisation.^60^ Schwann cells, which myelinate peripheral axons, are activated when TLR2 recognises pathogens and subsequently produce chemokines and cytokines including proinflammatory TNF‐α and IL‐6. Activation of TRPA1 in Schwann cells maintains pain sensation.^59^ In the CNS, activated astrocytes produce proinflammatory molecules including CXCL1 and CCL2 that sensitise dorsal horn neurons,^60^ and intrathecal injection of TNF‐α–activated astrocytes induces pain.^61^ Factors secreted upon afferent pain signalling such as ATP, calcitonin gene‐related peptide (CGRP) and MMP activate microglia.^60^ Microglial activation is typically early and transient compared with astrocytic activation, and spinal cord microglia produce proinflammatory cytokines that also sensitise dorsal horn neurons to pain.^57^

Keratinocytes secrete TSLP and endothelin‐1 (ET‐1), thus activating nerves, inducing itch and disrupting the skin barrier function. Keratinocytes also secrete NGF, which leads to hyperinnervation, increased sensitivity and barrier dysfunction.^46^, ^62^ Proinflammatory IL‐33 secreted by keratinocytes stimulates mast cells to produce numerous factors including TNF‐α and prostaglandin D2.^51^ Mast cells also undergo degranulation in response to sensory neuron‐derived substance P through the neurokinin receptor and Mas‐related G protein–coupled receptor (Mrgpr)X2.^62^ Macrophages express NGF and can respond to neuropeptides and thus are part of the bidirectional cross‐talk between the immune and nervous systems.^62^ Activated basophils show increased production of leukotriene C4, which directly activates sensory neurons via the cysteinyl leukotriene receptor 2 (CysLTR2) that acts via TRPV1 and TRPA1, leading to mast cell–dependent and basophil‐dependent acute itch (i.e. itch flares).^63^, ^64^ Thus, multiple cell types, including but not limited to immune and nerve cells, play roles in chronic stages of the pathology of AD (Figure 1).

### Neuroinflammation and the role of cytokines in increasing itch sensitivity via neurotrophism

Neuroinflammation includes trafficking of leukocytes into the CNS and peripheral nervous system (PNS), roles for satellite glial cells and secretion of proinflammatory molecules, leading to skin barrier dysfunction, which in turn induces or aggravates itch and pain.^7^, ^65^

Several cytokines increase sensitivity to stimuli by inducing branching and growth of sensory afferents (i.e. neurotrophism). Growth of sensory nerves including dermal neuropeptide‐positive fibres occurs in response to IL‐31^66^ and IL‐13.^15^ IL‐31 may also increase sensitivity to subthreshold stimuli and induce itch.^21^ In mice with transgenic expression of IL‐22 in the skin, GRP‐positive neurons in the DRG that innervated the skin were increased compared with wild‐type mice, and these mice showed intense scratching.^30^ Abnormal plasticity of peripheral sensory neurons contributes to the sensory perception of pain and itch and is present in patients with chronic pruritus, including those with AD; however, this was not because of hyperinnervation.^7^, ^67^

## Other molecules involved in itch and pain

### PAR‐2

Sensory nerve cells and keratinocytes express PAR‐2, which is cleaved by proteases released by degranulated mast cells.^68^ These effects are mediated by substance P and CGRP that are expressed by DRG neurons. Tryptase and PAR‐2 are increased in the skin of patients with AD,^69^ and PAR‐2 overexpression in keratinocytes is sufficient to induce lesions resembling AD.^70^ PAR‐2 activates TRPV4 channels in DRG neurons^71^ and NFκB in keratinocytes.^72^ Thus, following release of proteases by mast cells, cleaved PAR‐2 induces CGRP‐ and substance P–dependent neurogenic inflammation, itch and pain in patients with AD in a manner that also involves signalling via NFκB.^68^, ^71^, ^72^ Exogenous trigger factors such as allergens (house dust mite allergens) and endogenous trigger factors can activate PAR‐2, thereby contributing to itch and/or pain.^70^ These exogenous and endogenous factors are poorly understood.

### Ion channels

Neuronal TRP calcium ion channels modulate neuroimmune interactions and mediate itch and pain.^19^ TRPA1 and TRPV1 are major mediators of IL‐31–induced itch,^19^ and a TRPV1 antagonist attenuates AD and itching.^73^ TRP channels can be activated by bacteria such as *Staphylococcus aureus*, thereby inducing itch and pain in patients with AD.^74^ Chemokines are involved in itch and pain owing to activation of chemokine receptors, which are G protein–coupled receptors (GPCRs).^75^ The chemokines CXCL1 and CXCL2 modulate TRPV1 and reduce TRPV1 desensitisation in DRGs.^75^ IL‐13 activates TRPA1, leading to itch. TRPA1 is increased in dermal sensory fibres, DRGs, mast cells and keratinocytes and is involved in pain sensation.^15^ TRPV3 is expressed in keratinocytes and is overactive in AD model mice. Blocking TRPV3 attenuates itch and AD.^76^ Other ion channels involved in cutaneous itch and pain include acid‐sensing ion channels,^77^ potassium channels^78^ as well as NaV1.7, NaV1.8 and NaV1.9 sodium channels.^79^ Thus, ion channels play a role in itch and pain signalling.

### Neurotransmitters

Multiple neurotransmitters play a role in itch and pain signalling. Neuropeptide Y reduces IL‐31–mediated itch via presynaptic inhibition of afferent neurons.^80^ Substance P and NGF promote itch. Stimulated nerve fibres, which secrete substance P, vasoactive intestinal peptide and somatostatin, activate mast cells, which regulate nerve function. This positive feedback loop is mast cell dependent and TRPA1 dependent.^15^ Substance P is increased in AD and induces production of IFN‐γ, IL‐4, IL‐10 and TNF‐α, and treatments targeting substance P may be useful for AD.^81^ TNF‐α is also increased in AD^46^ and potentiates TSLP‐dependent calcium ion flow in DRGs, increasing the sensation of itch and indicating synergism between these cytokines.^46^ In addition to direct induction of itch, IL‐31 increases the secretion of BNP in DRGs and the skin, leading to increased secretion of other cytokines, chemokines and proteases in the skin and thus coordinating signalling that evokes itch.^45^ ET‐1 induces secretion of BNP and activates sensory neurons, various immune cells and keratinocytes.^46^ BNP induces itching, and inhibition of the BNP receptor, natriuretic peptide receptor 1, attenuates itch.^82^ Neuronal sensory fibres and eosinophils express brain‐derived neurotrophic factor (BDNF), the levels of which are higher in the skin of patients with AD than in patients with normal skin.^83^ Eosinophil‐derived BDNF appears to play a role in inducing branching of DRG neurons.^83^ BDNF promotes neurite outgrowth via JAK/STAT signalling. Thus, various secreted molecules work together to facilitate itch and afferent sensitivity in patients with AD.

### G protein–coupled receptors

Several GPCRs also mediate itch and pain, including Mrgpr family members and PAR‐2, as mentioned previously.^68^, ^72^ Neuropeptide Y,^80^ mu opioids,^84^ prostaglandin D_2_ ^85^ and substance P signal through GPCRs.^86^ The G protein–coupled oestrogen receptor modulates the effects of a TNF‐α–induced protein called A20 that is increased by formononetin, which attenuates AD.^87^ TRPA1, which is involved in itch and pain sensation, is activated downstream of GPCRs.^15^ Activation of GPCRs in sensory satellite glia directly modulates neuronal activity and induces analgesia.^65^ Thus, GPCRs are crucially involved in itch and pain signalling in AD.

## Itch, pain and the afferent nervous system

The afferent nervous pathways for itch and pain are distinct but also overlap and involve both the PNS and CNS.^88^, ^89^ Different pruritogens activate specific groups of afferent neurons that express their cognate receptor (e.g. substance P binds MrgprX, and the PAR‐2 agonist peptide binds PAR‐2).^90^

In the PNS, itch is mediated by C‐fibres and Aδ fibres, and pain is mediated by Aβ, Aδ and C‐fibres.^91^ Other cells are also involved in itch and pain such as Merkel cells, which are present in the basal portion of the epidermis and transmit touch and pressure sensations to sensory fibres.^91^ Itch‐specific small‐diameter sensory neurons in mice that directly detect pruritogens express MrgprA3, MrgprC11 and MrgprD.^89^ However, neurons that respond to both pain and itch have also been identified.^89^ RNA‐sequencing analysis has led to identification of multiple subtypes of sensory neurons, including multiple itch pathways, one of which involves IL‐31 and leukotrienes.^92^ Differences have been identified among mouse, human and non‐human primate DRGs.^93^, ^94^, ^95^ A subset of itch‐selective DRGs was identified with BNP expression,^96^ highlighting the differences among species.

In the CNS, itch circuits and pain circuits are present in the spinal cord and brain.^47^ Peripheral itch‐specific sensory neurons project to GRP receptor‐positive neurons in the spinal cord.^89^ A common population of dorsal horn spinal cord neurons responds to pain and itch stimuli.^19^ The sensation of itch or pain may depend on population coding in which the combination of fibres that are activated determines the quality (itch or pain) of the sensation^88^, ^97^ and/or on higher brain processing of the signals downstream of the primary sensory neurons.^19^ The hippocampus, amygdala and hypothalamus are activated by both pain and itch sensations.^89^

More than half of patients with AD report skin pain.^98^ In patients with AD, pain is modulated by IL‐12 and IL‐18 (which are upregulated in AD),^52^ prostaglandins, leukotrienes and MMPs.^99^, ^100^ Additional pain mediators include bradykinin, ATP, P2X purinoceptor 3 agonists, proteases, TLR agonists, cytokines (IL‐1, IL‐6, IL‐8, IL‐17, IL‐33, TSLP and TNF‐α), neurotrophins including NGF, ET‐1, CGRP, MrgprX1, MrgprX2 and MrgprD and ion channels (TRPVs, TRPA1, acid‐sensing ion channels, potassium ions and sodium ions).^60^, ^77^, ^101^, ^102^, ^103^, ^104^, ^105^, ^106^ The activity of many of these molecules is mediated by various populations of primary afferent fibres (e.g. peptidergic and nonpeptidergic) that synapse in different layers of the dorsal horn of the spinal cord. Interneurons in the dorsal horn modulate the signal travelling from spinal cord projection neurons to higher brain regions.^107^

Another mechanism of chronic itch and pain in skin involves descending central inhibition in which neural pathways from the brain inhibit pain or itch sensation of a normally nonpainful or non‐itch‐invoking stimulus. This system is impaired in patients with chronic itch.^7^, ^67^ Impaired descending central inhibition involves the spinal cord, brain stem and cortex and leads to central sensitisation, producing chronic pain and chronic itch^7^ induced by stimuli such as touch, clothing or warmth that normally do not induce these sensations.^108^ Thus, itch and pain in patients with AD result from aberrant cytokine expression, subsequent effects on the CNS and PNS and impaired neurologic function.

## Neuroimmune cross‐talk controlling itch and pain in AD

The immune system and nervous system show extensive bidirectional cross‐talk. The peripheral sensory and autonomic nervous system, which innervates the skin, has cross‐talk with mast cells, DCs, Th2 cells and other immune cells.^20^ Activated neurons release neurotransmitters and neuropeptides that affect innate and adaptive immune cells.^20^ In turn, immune cells release cytokines that activate peripheral nerves that mediate itch.^20^ Neuropeptide Y, which is expressed by dorsal horn interneurons and acts through the inhibitory Y_2_ receptor expressed on the central terminals of primary afferents, decreases IL‐31‐mediated itch.^80^ IL‐31 and TSLP are also neuroimmune links.^11^, ^19^ Mast cells, eosinophils and basophils interact with peripheral nerves to mediate skin inflammation, pain and itch in patients with AD via IL‐4, IL‐13, IL‐31, OSM, neuropeptides and substance P.^25^, ^38^ Thus, the bidirectional communication between the nervous system and immune system can work to amplify the pathologic state in patients with AD (Figure 1).

## Therapeutic approaches in AD

### JAK signalling

**Figure 2:**
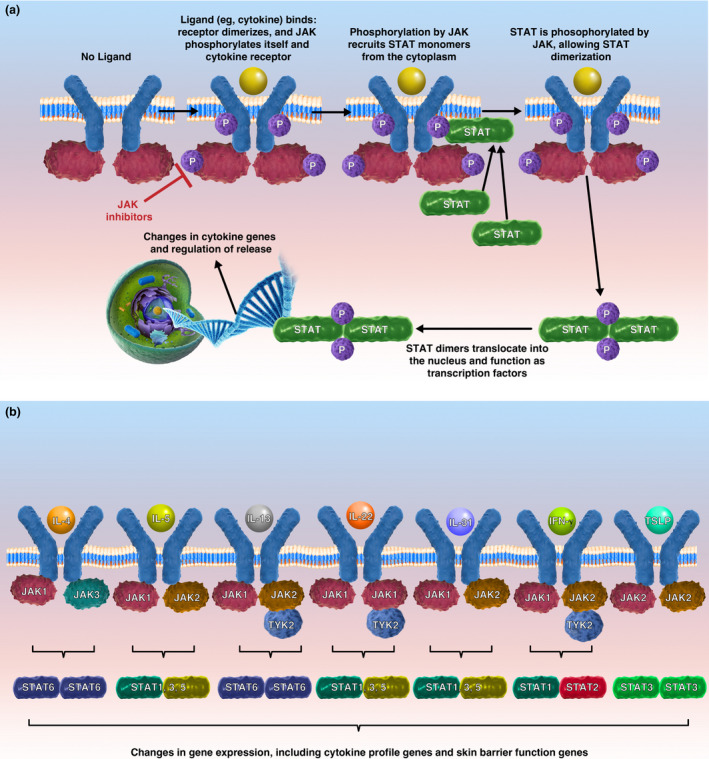
JAK/STAT signalling. **(a)** Upon ligand binding (e.g. cytokines), the cytokine receptor dimerises, and JAK phosphorylates itself and the intracellular region of the cytokine receptor. Phosphorylation of the receptor recruits STAT monomers from the cytoplasm. JAK phosphorylates STAT, which then forms a dimer. STAT dimers translocate into the nucleus where they act as transcription factors and impact gene expression, including expression of cytokine genes involved in inflammation, epidermal skin barrier, itch and pain. JAK inhibitors interact with the P‐loop of the JAK kinase domain,^148^ and hence inhibit events downstream of JAK phosphorylation (JAK, Janus kinase; P, phosphorylation; STAT, signal transducer and activator of transcription). **(b)** Key cytokines in atopic dermatitis and the JAK and STAT molecules that mediate signalling (IL, interleukin; IFN, interferon; TSLP, thymic stromal lymphopoietin).

**Figure 3:**
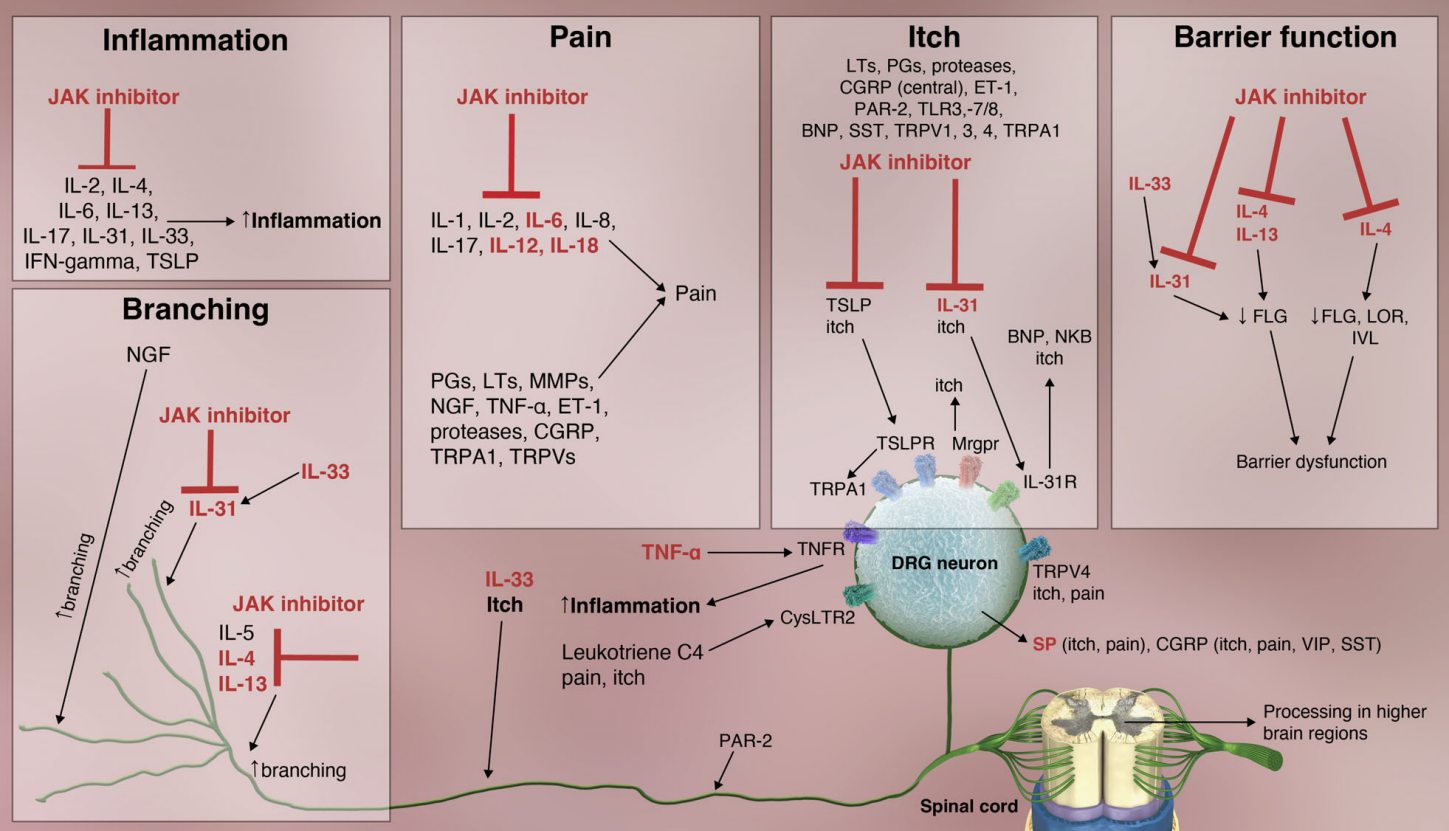
Effects of JAK inhibitors on neuroimmune circuits and barrier dysfunction in atopic dermatitis. Cytokines in red/bold text are upregulated in atopic dermatitis (BNP, brain natriuretic peptide; CGRP, calcitonin gene‐related peptide; CysLTR2, cysteinyl leukotriene receptor; DRG, dorsal root ganglion; ET‐1, endothelin 1; FLG, filaggrin; IFN, interferon; IL, interleukin; IL‐31R, IL‐31 receptor; IVL, involucrin; JAK, Janus kinase; LOR, loricrin; LT, leukotriene; MMP, matrix metalloproteinase; MrgprX1, Mas‐related G protein–coupled receptor X1; MrgprX2; Mas‐related G protein–coupled receptor X2; MrgprD, Mas‐related G protein–coupled receptor‐D; NGF, nerve growth factor; NKB, neurokinin B; PAR‐2, proteinase‐activated receptor 2; PG, prostaglandin; SP, substance P; SST, somatostatin; TLR, toll‐like receptor; TNF, tumor necrosis factor; TNFR, tumor necrosis factor receptor; TRPA1, transient receptor potential ankyrin 1; TRPV, transient receptor potential vanilloid; TSLP, thymic stromal lymphopoietin; TSLPR, thymic stromal lymphopoietin receptor; VIP, vasoactive intestinal peptide).

Multiple cytokines implicated in AD signal through JAK and various STATs (Figure 2).^8^ For example, IL‐4, IL‐13, IL‐31, TSLP, IL‐5, IL‐22 and IFN‐γ signal through JAK1.^109^, ^110^, ^111^, ^112^ As reviewed above, JAK signalling also plays a role in cell signalling pathways in peripheral nerves that contribute to itch and pain, thus providing a rationale for the use of JAK inhibitors (JAKi) to treat AD (Figure 3). IL‐4 and IL‐13, signalling through all members of the JAK family and STAT6 homodimers, decrease expression of genes involved in keratinocyte differentiation^111^, ^113^ and that of the antimicrobial genes human beta‐defensins (*HBD*)‐2 and *HBD*‐3.^114^ IL‐4 also affects MHC class II expression, and IL‐4 and IL‐13 affect Th2 cell differentiation (via changes in GATA‐3 expression) and IgE class switching.^114^

IL‐4 signals through JAK1 and JAK3 and STAT6 homodimers, resulting in downregulation of the expression of several genes in the epidermal differentiation complex including filaggrin (*FLG*), loricrin (*LOR*) and involucrin (*IVL*).^21^, ^114^ The use of JAKi may mitigate these effects. For example, the JAK1/3 inhibitor, tofacitinib, increases expression of *DSC1*, *FLG* and *KRT1*.^115^

IL‐31 signals through JAK1 and JAK2^109^ and induces changes in pro‐opiomelanocortin, which regulates itch in AD,^116^ and downregulates filaggrin expression, resulting in disrupted barrier function.^35^ IL‐31 also leads to increased secretion of other cytokines, chemokines and proteases in the skin to coordinate signalling that leads to itch.^45^

IL‐33 does not signal through JAK, but induces secretion of IL‐31, TNF‐α and CXCL8.^117^, ^118^ Enhanced JAK signalling via elevated proinflammatory cytokines and subsequent changes in gene expression downstream of JAK signalling propagate this dysregulated immune response and exacerbate the abnormal cytokine profile.

In addition to direct effects, JAKi can indirectly suppress cytokines such as IL‐17 that do not signal through JAK/STAT by inhibiting other JAK/STAT‐dependent cytokines such as IL‐23.^8^ Favourable alterations in gene expression by JAKi are also expected to lead to improvements in barrier function, pain and itch. Decreased expression of genes in the epidermal differentiation complex by IL‐4 decreases epidermal barrier function,^114^ and JAKi are expected to help restore barrier function by blocking these decreases (Figure 3). Descending central inhibitory pathways are abnormal in patients with AD and in those with rheumatoid arthritis. JAKi, which are used to treat patients with rheumatoid arthritis, attenuate central pain processing that is overactive owing to chronic inflammation.^119^ Thus, the use of JAKi in patients with AD is expected to restore the cytokine profile to a more normal state, improve barrier function and itch and decrease pain by affecting CNS processing.

### Preventive and other approaches

The disease course of AD is often relapsing and remitting.^120^ The goals of treatment include prevention of flares to help control pain and itch and to repair and maintain a functional skin barrier.^120^, ^121^ Preventive treatments for AD include various topical therapies as well as systemic options including phototherapy.^122^ Topical treatment options include topical steroids, calcineurin inhibitors, phosphodiesterase‐4 inhibitors, JAK/STAT inhibitors and wet wrap therapy with topical steroids.^2^, ^120^ Occasionally, antibiotics or antiseptics are used to treat infected lesions.^121^

### Clinical overview and differentiation

Patient‐reported outcomes are important for selecting therapy for AD.^3^, ^123^ Patients with uncontrolled disease report higher burdens of itch, psychological comorbidities and sleep deprivation.^124^ Inadequate disease control is common among those with moderate‐to‐severe AD, highlighting the need for more effective treatments.^1^, ^124^ Several inhibitors of the JAK/STAT signalling pathway, both oral and topical, have been developed or are currently under development (Table 2).^2^ In patients with inadequate disease control, systemic options that target multiple cytokines in AD may afford more effective disease control than topical interventions. Here, we explore the rationale for emerging systemic treatment options for AD by considering all signs and symptoms.

**Table 2:** Current and potentially future treatment options for atopic dermatitis

Drug	Target(s)	Mode of administration	Development status	Comment	References
Baricitinib	JAK1/2	Oral	Approved in the European Union for moderate‐to‐severe AD	Increase in number of patients achieving EASI‐50 at 16 weeks; treatment effect seen at 4 weeks	12, 131
Abrocitinib	JAK1	Oral	Approved in several countries for moderate‐to‐severe AD	Improvements in EASI, IGA, EASI‐75, EASI‐90 and PP‐NRS	132, 133, 134, 137, 138, 139
Upadacitinib	JAK1	Oral	3 phase 3 trials completed; approved in the European Union and United States	About half of patients achieved EASI‐75 and IGA 0/1	12, 135, 136
ASN002	JAK1, 2, 3, TYK and SYK	Oral	Phase 1b	Improvement in itch starting on day 8; 83–100% of patients achieved EASI‐50 at the 2 highest doses	12
Delgocitinib	All JAKs	Topical	Approved in Japan for AD; FDA Fast‐Track Designation for chronic hand eczema	Significant improvements in modified EASI	125
Tofacitinib	JAK1/3	Topical	Phase 2a	Off‐label; significant changes in EASI PGA, BSA and pruritus	143
Ruxolitinib	JAK1/2	Topical	Approved for mild‐to‐moderate AD in the United States	Off‐label; IGA treatment success achieved; improvements in itch	126, 144
Roflumilast	PDE4	Topical	Phase 2a	No significant changes in SCORAD, TEWL or pruritus	145
Tapinarof	AHR	Topical	Phase 2b	Week 12 IGA responses, EASI scores, pruritus and POEM improved; %BSA reduced	146
Dupilumab	IL‐4Rα	Subcutaneous injectable	FDA approved	≤ 50% decrease in PP‐NRS at week 16; itch improvement as early as 1–3 days	141
Tralokinumab	IL‐13	Subcutaneous injectable	Approved in the European Union and United States for moderate‐to‐severe AD	Improvements in IGA, pruritus, sleep interference, DLQI, POEM and SCORAD	129, 130, 147
Nemolizumab	IL‐31	Subcutaneous injectable	Phase 2	Significant changes in pruritus at week 12; 70–80% change in pruritus score after 64 weeks	12, 36
Tezepelumab	TSLP	Subcutaneous injectable	Phase 2	Non‐statistically significant improvement in EASI‐50 for tezepelumab group vs placebo	12, 43
GBR 830	OX40	Intravenous infusion	Phase 2	Increase in number of patients achieving EASI‐50	12
Etokimab	IL‐33		Phase 2a	All patients achieved EASI‐50 or better; however, primary endpoint not met in a phase 2b trial	12
Fezakinumab	IL‐22	Intravenous infusion	Phase 2a	Improvements in SCORAD and IGA in patients with severe, but not moderate, AD; improvements in %BSA	12

%BSA, percent of body surface area; AD, atopic dermatitis; AHR, aryl hydrocarbon receptor; DLQI, Dermatology Life Quality Index; EASI, Eczema Area and Severity Index; EASI‐50, −75 and −90, 50%, 75% and 90%, respectively, improvement in EASI score from baseline; FDA, Food and Drug Administration; IGA, Investigator’s Global Assessment; IL, interleukin; JAK, Janus kinase; PDE, phosphodiesterase; POEM, Patient‐Oriented Eczema Measure; PP‐NRS, Peak Pruritus Numerical Rating Scale; SCORAD, SCORing Atopic Dermatitis; SYK, spleen tyrosine kinase; TEWL, transepidermal water loss; TSLP, thymic stromal lymphopoietin; TYK, leukocyte receptor tyrosine kinase.

### Topical treatment options

The topical pan‐JAKi, delgocitinib (JTE‐052), improves barrier function by blocking IL‐4/IL‐13/JAK/STAT3/6–mediated signalling and subsequent chemokine expression by keratinocytes.^113^ Delgocitinib is effective in improving the Eczema Area and Severity Index (EASI) score.^125^ The topical JAKi ruxolitinib is approved for the treatment of mild‐to‐moderate AD.^126^ The topical aryl hydrocarbon receptor (AHR) mediates keratinocyte differentiation. Tapinarof, an AHR modulator, increases FLG and LOR expression and also induces IL‐24, which activates JAK/STAT and decreases FLG and LOR expression. Data support a new strategy for treating AD using both AHR modulators and JAKi.^127^

### Systemic treatment options

Currently, approved biologic therapies for AD include dupilumab, a monoclonal antibody that targets IL‐4Rα, which is activated by IL‐4 and IL‐13,^128^ and tralokinumab, which targets IL‐13.^129^, ^130^ Treatment with dupilumab leads to favourable changes in gene expression in patients with AD, including effects on inflammatory mediators, barrier‐related genes, chemokines and cytokines.^128^ These changes parallel clinical improvements in patients receiving dupilumab.^128^ Other injectable systemic therapies are currently in clinical trials for moderate‐to‐severe AD targeting IL‐13 alone or IL‐31.^16^, ^36^

Baricitinib, which is approved in the European Union for moderate‐to‐severe AD, is an orally available JAK1/2 inhibitor.^12^ A phase 3 trial showed that at week 16, the proportion of patients with moderate‐to‐severe AD who achieved a 75% improvement in the EASI score from baseline (EASI‐75) and a validated Investigator's Global Assessment for Atopic Dermatitis response (vIGA‐AD) was significantly higher in the baricitinib 2‐mg group than the placebo group.^131^ Upadacitinib and abrocitinib are orally available selective JAK1 inhibitors.^12^ Abrocitinib is approved in several countries for the treatment of patients with moderate‐to‐severe AD in who are candidates for systemic therapy.^132^, ^133^, ^134^ Upadacitinib is approved by the European Medicines Agency and the US Food and Drug Administration (FDA). Three phase 3 trials with upadacitinib showed that at week 16, the proportions of patients who had achieved EASI‐75 and a vIGA‐AD response were significantly higher in the upadacitinib 15‐ or 30‐mg groups (as monotherapy or with topical corticosteroids) than in the placebo group.^135^, ^136^ In patients with moderate‐to‐severe AD, abrocitinib showed acceptable safety and good efficacy, including a higher proportion of patients in the 100‐ and 200‐mg abrocitinib groups achieving EASI‐75, an Investigator’s Global Assessment response,^137^, ^138^, ^139^ and a Peak Pruritus Numerical Rating Scale response^138^ compared with placebo at week 12. ASN002 is an orally available inhibitor of all four JAK family members and spleen tyrosine kinase inhibitor (SYK), and showed rapid improvement in itching and EASI‐50 scores.^2^

Thus, emerging clinical data show that JAKi are an effective systemic option for patients with moderate‐to‐severe AD, providing rapid improvement in itch symptoms, and overall, JAKi have a favourable safety profile.^140^ These data are consistent with a pivotal role for the JAK/STAT signalling pathway in mediating changes in gene expression that inhibit molecular mechanisms of host immune dysregulation and itch and pain response in patients with AD.

## Conclusions and future perspectives

The main symptoms of AD are itch and pain, which significantly affect patients’ quality of life. Primary afferent sensory nerves play a key role by transmitting itch and pain to the CNS; in addition, axon reflex mechanisms of those nerves release neuromediators in the skin of patients with AD, thereby aggravating inflammation, itch and pain. Recent evidence indicates that these neuroimmune circuits are induced by and also change the cytokine profile in AD, thus modulating itch and pain in those patients. Thus, cytokines affect one another and also mediate the cross‐talk between the immune and afferent nervous system via neuronal cytokine receptors. Many cytokine receptors signal through JAK, and several JAKi are in clinical development for the treatment of patients with AD. JAKi modulate gene expression to restore normal cytokine profiles, which results in downstream effects on neuroimmune cross‐talk that reduce itch and pain. Other sensations such as burning and hypersensitivity (i.e. alloknesis) caused by neurotrophic effects require more detailed investigation. Important remaining questions are as follows: (1) which cytokines are the most important in itch and/or pain; (2) which JAK/STAT pathways are the most critical to control itch and pain in AD; and (3) which JAKi and biologics are the most significant to successfully and sustainably suppress cytokine‐mediated itch and pain in AD? Answering these questions will lead to optimal control of these debilitating symptoms in patients with AD.

## Conflict of interest

SGK is an advisory board member/consultant for Pfizer Inc., AbbVie, Celldex Therapeutics, Galderma, Incyte, Regeneron Pharmaceuticals and Kiniksa Pharmaceuticals and has served as an investigator for Pfizer Inc., Galderma, Kiniksa Pharmaceuticals and Sanofi. LM is an advisory board member/consultant for Pfizer Inc., AbbVie, Bayer, Galderma, LEO Pharma, Lilly, Novartis and Sanofi; has served as an investigator for Pfizer Inc., Abbvie, Almirall, Dermira, Galderma, Kiniksa Pharmaceuticals, Lilly, Novartis, Sanofi and Trevi; and has received grants from Beiersdorf, Clarins and Johnson & Johnson. CC is an employee and shareholder of Pfizer Inc. MS is an advisory board member/consultant/speaker for Pfizer Inc., AbbVie, Algorithm, Almirall, Avon, Bayer, Baiersdorf, Celgene, Chugai, Galderma, GSK, Novartis, Incyte, Kiniksa Pharmaceuticals, LEO Pharma, Eli Lilly, Janssen, Johnson & Johnson, L’Oreal, Maruho, MenloTx, Mitsubishi, Novartis, Pierre Fabre, Qatar Pharm, Regeneron, Sanofi, Toray, Vertex and Zymogenetics; and has received grant funding from Pfizer Inc., AbbVie, Almirall, Avon, BMS, Chugai, Galderma, Janssen, Johnson & Johnson, Pierre Fabre, Novartis, L’Oreal, Maruho, Sanofi, Toray and Zymogenetics.

## Author contributions

**Shawn G Kwatra:** Conceptualization; Writing – original draft; Writing – review & editing. **Laurent Misery:** Writing – review & editing. **Claire Clibborn:** Writing – review & editing. **Martin Steinhoff:** Conceptualization; Writing – original draft; Writing – review & editing.
